# A multicenter cross-sectional French study of the impact of COVID-19 on neuromuscular diseases

**DOI:** 10.1186/s13023-021-02090-y

**Published:** 2021-10-26

**Authors:** Lucie Isoline Pisella, Sara Fernandes, Guilhem Solé, Tanya Stojkovic, Céline Tard, Jean-Baptiste Chanson, Françoise Bouhour, Emmanuelle Salort-Campana, Guillemette Beaudonnet, Louise Debergé, Fanny Duval, Aude-Marie Grapperon, Marion Masingue, Aleksandra Nadaj-Pakleza, Yann Péréon, Frédérique Audic, Anthony Behin, Diane Friedman, Armelle Magot, Jean-Baptiste Noury, Sarah Souvannanorath, Karim Wahbi, Jean-Christophe Antoine, Kévin Bigaut, Jean-Philippe Camdessanché, Pascal Cintas, Rabab Debs, Caroline Espil-Taris, Laurent Kremer, Thierry Kuntzer, Pascal Laforêt, Vincent Laugel, Martial Mallaret, Maud Michaud, Sylvain Nollet, Juliette Svahn, Savine Vicart, Rocio Nur Villar-Quiles, Isabelle Desguerre, David Adams, Sandrine Segovia-Kueny, Géraldine Merret, Elhadi Hammouda, Annamaria Molon, Shahram Attarian

**Affiliations:** 1 Filnemus, AP-HM, Marseille, France; 2 Department of Epidemiology and Health Economics, AP-HM, Marseille, France; 3 Department of Neurology and Neuromuscular Disorders, Reference Center for Neuromuscular Disorders AOC, University Hospitals of Bordeaux (Pellegrin University Hospital), place Amélie-Raba-Léon, 33076 Bordeaux, France; 4 Reference Center for Neuromuscular Disorders Nord/Est/Île-de-France, Sorbonne Université, AP-HP, Hôpital Pitié-Salpêtrière, Inserm UMR_S 974, Paris, France; 5 CHU de Lille, Inserm U1171, Reference Center for Neuromuscular Disorders Nord/Est/Île-de-France, Lille, France; 6 Neurology Department, Reference Center for Neuromuscular Diseases ‘Nord-Est-Ile de France’, University Hospitals of Strasbourg, Strasbourg, France; 7 ENMG Unit, Reference Center for Neuromuscular Diseases, University Hospitals of Lyon (Neurologic Hospital Pierre Wertheimer), Lyon, France; 8 Reference Center for Neuromuscular Diseases and ALS, Timone University Hospital, Aix-Marseille University, ERN-Center, Marseille, France; 9 Clinical Neurophysiology Unit, Reference Center for Neuromuscular Disease, University Hospital of Bicetre, Le Kremlin Bicêtre, France; 10 Reference Center for Neuromuscular Diseases, Filnemus, ERN Euro-NMD, CHU Nantes, Nantes, France; 11 Reference Center for Neuromuscular Diseases, Neuropediatric Unit Timone University Hospital, Marseille, France; 12 Neurology Department, Nord/Est/Île-de-France Neuromuscular Reference Center, Raymond-Poincaré Teaching Hospital, AP-HP, Garches, France; 13 INSERM U1179, END-ICAP, Versailles-Saint-Quentin-en-Yvelines University, Université Paris Saclay, Montigny-le-Bretonneux, France; 14 Reference Center for Neuromuscular Diseases AOC, University Hospital of Brest, Brest, France; 15 Reference Center for Neuromuscular Diseases, Henri Mondor University Hospital, Assistance Publique - Hôpitaux de Paris, Créteil, France; 16 AP-HP, Cochin Hospital, Cardiology Department, FILNEMUS, Reference Center for Neuromuscular Diseases Nord/Est/Île-de-France, Paris-Descartes, Sorbonne Paris Cité University, 75006 Paris, France; 17 INSERM Unit 970, Paris Cardiovascular Research Center (PARCC), Paris, France; 18 Department of Neurology, Reference Center for Neuromuscular Diseases, University Hospital of Saint-Etienne, Saint-Etienne, France; 19 Department of Neurology, Reference Center for Neuromuscular Diseases, University Hospitals of Toulouse (Purpan Hospital), Toulouse, France; 20 Clinical Neurophysiology Department, Hôpital Pitié-Salpêtrière, APHP Paris VI Université, Paris, France; 21 Department of Pediatric Neurology, Neuromuscular Center, CHU Bordeaux, Bordeaux, France; 22 Nerve-Muscle Unit, Neurology Service, Department of Clinical Neurosciences, Lausanne University Hospital (CHUV), University of Lausanne, Lausanne, Switzerland; 23 Department of Pediatrics, Strasbourg University Hospital, Strasbourg Cedex, France; 24 Department of Neurology, Competence Center for Neuromuscular Diseases, University Hospital Centre Grenoble Alpes, CS 10217, 38043 Grenoble Cedex 9, France; 25 Department of Neurology, Nancy University Hospital, Nancy, France; 26 Clinical Neurology-Electrophysiology Department, University Hospital (CHRU) Besançon, Besançon, France; 27 Reference Center for Neuromuscular Disorders Nord/Est/Île-de-France, Pediatric Neurology Department, Necker-Enfants-Malades Hospital, AP-HP, Paris, France; 28 Department of Neurology, University Hospital of Bicêtre, Le Kremlin-Bicêtre, France; 29 AFM-Téléthon, Evry, France

**Keywords:** Neuromuscular diseases, COVID-19, Risk factor, Prognosis

## Abstract

**Background:**

Due to their health condition, patients with neuromuscular diseases (NMD) are at greater risk of developing serious complications with COVID-19. The objective of this study was to analyze the prevalence of COVID-19 among NMD patients and the risk factors for its impact and severity during the first wave of the pandemic. Clinical data were collected from NMD-COVID-19 patients, between March 25, 2020 and May 11, 2020 in an anonymous survey carried out by expert physicians from the French Health Care Network Filnemus.

**Results:**

Physicians reported 84 patients, including: 34 with myasthenia gravis, 27 with myopathy and 23 with neuropathy. COVID-19 had no effect on NMD for 48 (58%) patients and 48 (58%) patients developed low COVID-19 severity. COVID-19 caused the death of 9 (11%) NMD patients. Diabetic patients were at greater risk of dying. Patients with diabetes, hypertension or severe forms of NMD had a higher risk of developing a moderate or severe form of COVID-19. In our cohort, corticosteroids and other immunosuppressants were not significantly associated with higher COVID-19 severity for acquired NMD.

**Conclusion:**

During this period, a small percentage of French NMD patients was affected by COVID-19 compared to the general French population and COVID-19 had a limited short-term effect on them. Diabetes, hypertension and a severe degree of NMD were identified as risk factors of unfavorable outcome following COVID-19. Conversely, in our cohort of patients with acquired NMD, corticosteroids or other immunosuppressants did not appear to be risk factors for more severe COVID-19.

## Background

Coronavirus-disease-2019 (COVID-19) is caused by coronavirus-2 (SARS-CoV-2). It quickly struck the entire world after its first appearance in China in December 2019 and had catastrophic consequences on populations and health systems worldwide [1]. In France, public health reports on September 21, 2021 declared that 6 956 848 patients had been infected, causing 116 050 deaths [2]. Although COVID-19 has great similarities to SARS and MERS, genome sequencing of SARS-CoV-2 has revealed that it is a novel coronavirus [3]. COVID-19 leads to a wide range of clinical signs including fever, fatigue, dry cough, headache, myalgia and anosmia [4, 5]. The symptoms and severity of COVID-19 depend on the patient’s age, health condition, sex and immune system [6].

In France, according to the National Data Bank of Rare Diseases (BNDMR), 50 000 patients suffer from neuromuscular diseases (NMD), a large group of rare diseases (300 forms listed to date) most often of genetic or autoimmune origin which affect both children and adults. As they can be under immunosuppressive and/or immunomodulator therapy, or subject to cardiac or respiratory failures, patients with NMDs are generally considered at high risk of developing serious complications with COVID-19 [6].

In 2014, the French Ministry of Health created the French Health Care Network for Rare NMDs named Filnemus (www.filnemus.fr). Filnemus is a structure with a vast territorial network comprising 71 expert centers [7]. The missions of Filnemus are to coordinate, promote and convene the various actions carried out in the field of rare NMDs.

Facing the COVID-19 public health emergency, the 71 expert centers of Filnemus, in collaboration with patient organizations involved with NMD, set up a national observatory to enable the collection of data for NMD patients with COVID-19. This national multicenter study aimed to determine the prevalence of COVID-19 for NMD patients and the risk factors for its impact and severity during the first French lockdown period.

## Results

### Cohort description

**Fig. 1:**
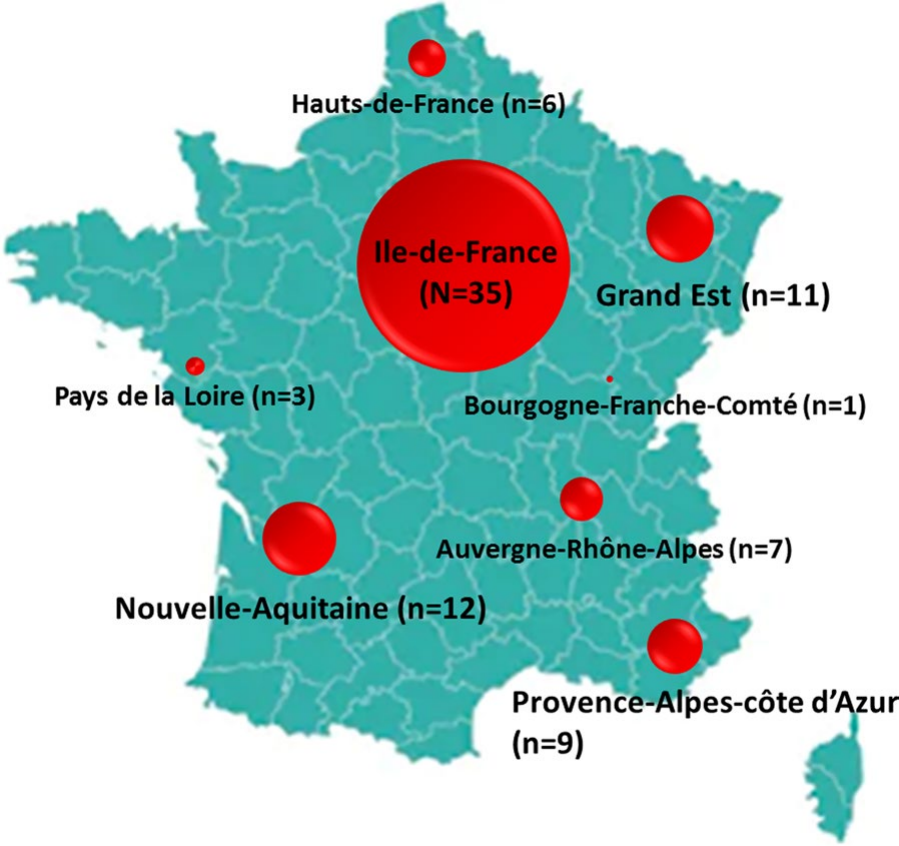
French geographical repartition of neuromuscular COVID-19 positive cases: size of red circle correspond to the number of neuromuscular COVID-19 positive cases per French region (*n* number of cases per region)

Data were collected from March 25, 2020 to May 11, 2020 during the first lockdown period in France. During this period, 84 patients followed up in the expert centers and with a diagnosis of definite or probable COVID-19 were reported by experts (Table 1). The cohort included 34 patients with myasthenia gravis, 27 with myopathy and 23 with neuropathy (Table 1). No cases of COVID-19 patients with spinal muscular atrophy were reported during this period. The geographical distribution by region is shown in Fig. 1.

**Table 1:** Listing of neuromuscular diseases for patients with COVID-19 (*n* number of patients)

Myasthenia	n	Myopathy	n	Neuropathy	n
Autoimmune myasthenia gravis	32	Becker muscular dystrophy	1	Anti-MAG neuropathy	1
Congenital myasthenic syndromes	2	Beta sarcoglycanopathy	1	Chronic Ataxic Neuropathy, Ophtamoplegia, IgM paraprotein, cold Agglutinins and Disialosyl antibodies	1
		Calpainopathy (LGMD2A)	3	Cerebellar Ataxia, Neuropathy, Vestibular Areflexia Syndrome	1
		Congenital muscular dystrophy	1	Charcot-Marie-Tooth disease	8
		Congenital myotonia (Becker)	1	Chronic demyelinating inflammatory polyradiculoneuritis	4
		Duchenne muscular dystrophy	3	Hereditary amyloid neuropathy	2
		Gamma sarcoglycanopathy	1	Mitochondrial cytopathy	1
		GNE myopathy	2	Multifocal motor neuropathy	2
		Inclusion body myositis	1	Neuromyotonia	1
		Limb-girdle muscular dystrophy	1	Sensory neuropathy	2
		Mitochondrial myopathy	1		
		Muscle channelopathy	1		
		Myofibrillary myopathy	1		
		Myositis	1		
		Type 1 myotonic dystrophy (Steinert)	4		
		Type 2 myotonic dystrophy (PROMM)	1		
		Type 3 glycogenosis	2		
		Unlabeled myopathy	1		
	34		27		23

Twenty-eight patients had respiratory failure, 16 patients had heart failure, 10 patients had diabetes, 11 patients had high arterial blood pressure, 8 patients suffered from obesity and 19 patients were over 65 years old (Table 2). The mean age of the patients was 50 years (range 18–94) and the female/male ratio was 1.47 (Table 2). The diagnosis of COVID-19 was established by PCR in 24 patients, based on thoracic CT-Scan results in 4 patients, by both PCR and thoracic CT-Scan in 20 patients, and by clinical signs highly suggestive of COVID-19 in 36 patients (Table 2). The prevalence of symptomatic COVID-19 was estimated at 0.0017. The hospitalization rate in medical units and intensive care unit (ICU) was respectively 0.27 and 0.14. Forty-eight patients received home care.

**Table 2:** Description of the cohort

	Total (n = 84) No. (%)
Age, years (mean ± SD) (min–max)	49.8 ± 18.70 (18–94)
Age > 65 years old	19 (22.9)
Gender (male)	34 (40.46)
Type of disease	
Myopathy	27 (40.5)
Neuropathy	23 (27.4)
Myasthenia	34 (40.5)
Origin of the disease	
Acquired	44 (53.0)
Genetic	39 (47.0)
Severity of neuromuscular disease^Ɨ^	
Low	30 (36.6)
Moderate	36 (43.9)
High	16 (19.5)
Mobility	
Walk	58 (70.7)
Walk with help	12 (14.6)
Wheelchair	12 (14.6)
Comorbidities	
Heart failure	16 (20.3)
Respiratory failure	28 (35.9)
Hypertension	11 (13.1)
Diabetes	10 (11.9)
Obesity	8 (9.5)
Medication^‡^	
Corticosteroids	14 (16.7)
Other immunosuppressants	17 (20.2)
Diagnosis of COVID-19	
PCR	24 (28.6)
Thoracic CT-Scan	4 (4.8)
PCR + Thoracic CT-Scan	20 (23.8)
Neither ^§^	36 (42.8)
Effect of COVID-19	
Without	48 (57.8)
Aggravating factor	26 (31.3)
Death	9 (10.8)
Severity of COVID-19	
Low	48 (57.8)
Moderate	23 (27.7)
High	12 (14.5)
Medical care	
Medical unit	23 (27.7)
Intensive care unit	12 (14.5)
Home care	48 (57.8)

*n* number of cases, *No. (%)* number of cases (% of cases)

^Ɨ^According to the physician

^‡^Prior to COVID-19

^§^Patients presenting clinical signs highly suggestive of COVID-19 (cough, fever, anosmia, hyposmia, skin signs, myalgia, headache, diarrhea, breathlessness, chest tightness)

### Impact of COVID-19 on NMD

For 48 (58%) NMD patients, COVID-19 had no effect on their pre-existing disease. COVID-19 caused worsening of symptoms in 26 (31%) NMD patients and death in 9 (11%) NMD patients. The death rate was therefore 0.11. Of the 9 deaths recorded only 2 patients had a low severe neuromuscular disease, one of whom was diabetic and suffered from hypertension. Four out of 9 of deceased patients presented respiratory failure, 1/9 presented cardiac failure, 5/9 presented diabetes, and 3/9 presented hypertension. Six out of 9 deceased patients presented at least two comorbidities such as respiratory or cardiac failure, hypertension, diabetes or being aged > 65 years. One patient was a 43-year-old man with myasthenia gravis and who presented no respiratory/cardiac failure, no comorbidity and a low degree of severity of neuromuscular disease. The cause of his death was acute respiratory distress syndrome.

Table 3 provides a description of the NMD cohort according to the impact of COVID-19 on NMD.

**Table 3:** Impact of COVID-19 on NMD

	Without effect (n = 48) No. (%)	Aggravating factor (n = 26) No. (%)	Death (n = 9) No. (%)	p value
Type of disease				0.716
Myopathy	17 (63)	8 (29.6)	2 (7.4)	
Neuropathy	15 (65.2)	6 (26.1)	2 (8.7)	
Myasthenia	16 (48.5)	12 (36.4)	5(15.2)	
Origin of the disease				0.322
Acquired	23 (53.5)	13 (30.2)	7 (16.3)	
Genetic	24 (61.5)	13 (33.3)	2 (5.1)	
Severity of neuromuscular disease^Ɨ^				0.170
Low	21 (70.0)	7 (23.3)	2 (6.7)	
Moderate	19 (54.3)	13 (37.1)	3 (8.6)	
High	6 (37.5)	6 (37.5)	4 (25.0)	
Mobility				0.110
Walk	35 (61.4)	20 (35.1)	2 (3.5)	
Walk with help	7 (58.3)	3 (25.0)	2 (16.7)	
Wheelchair	6 (50.0)	3 (25.0)	3 (25.0)	
Gender				0.079
Men	15 (44.1)	13 (38.2)	6 (17.6)	
Women	33 (67.3)	13 (26.5)	3 (6.1)	
Age > 65 yrs old	9 (47.4)	8 (42.1)	2 (10.5)	0.553
Respiratory failure	13 (48.1)	10 (37.0)	4 (14.8)	0.233
Heart failure	12 (75.0)	3 (18.8)	1 (6.2)	0.401
Obesity	0	7 (87.5)	1 (12.5)	0.001*
Hypertension	4 (36.4)	4 (36.4)	3 (27.3)	0.097
Diabetes	2 (20.0)	3 (30.0)	5 (50.0)	< 0.001*
Corticosteroids^‡^^,^^§^	9 (69.2)	2 (15.4)	2 (15.4)	0.394
Immunosuppressants other than corticosteroids^‡^^,^^§^	9 (60.0)	5 (33.3)	1 (6.7)	0.565

*n* number of cases, *No. (%)* number of cases (% of cases)

^Ɨ^According to the physician

^‡^Prior to COVID-19

^§^Statistical analysis have been performed only on populations with acquired disease

*p < 0.05 was statistically significant

Univariate analysis revealed that obesity (p = 0.001) and diabetes (p < 0.001) were significantly associated with the exacerbation of NMD (Table 3).

Diabetes, along with gender (p = 0.079), hypertension (p = 0.097) and degree of mobility (p = 0.110) were included in a multinomial logistic regression (obesity was excluded due to small numbers, which were insufficient to enable the model to converge to a final solution). Respiratory failure was not significant (p = 0.233) but it is a risk factor known to influence the patient's health condition following infection with SARS-CoV2 and was therefore included in the model. Compared to the reference category “without effect”, multinomial logistic regression showed that patients with diabetes (OR = 44.000; 95% CI 4.821–401.548, p = 0.001) had a higher risk of death following COVID-19.

Concerning medication, statistical analysis performed only on the population suffering from acquired and not hereditary forms of NMD showed that corticosteroids or other immunosuppressants were not significantly associated with the impact of COVID-19 on NMD (p = 0.394 and p = 0.565 respectively).

### Impact of NMD on the severity of COVID-19

For 48 (58%) NMD patients, the severity of COVID-19 was low. Moderate COVID-19 severity was reported in 23 (28%) NMD patients, while 12 (15%) NMD patients suffered from highly severe COVID-19. Table 4 provides a description of the NMD cohort according to the impact of NMD on the severity of COVID-19.

**Table 4:** Impact of NMD on COVID-19

	Low COVID-19 severity (n = 48) No. (%)	Moderate COVID-19 severity (n = 23) No. (%)	High COVID-19 severity (n = 12) No. (%)	p-value
Type of disease				0.095
Myopathy	16 (59.3)	8 (29.6)	3 (11.1)	
Neuropathy	12 (52.2)	10 (43.5)	1 (4.3)	
Myasthenia	20 (60.6)	5 (15.2)	8 (24.2)	
Origin of the disease				0.199
Acquired	24 (55.8)	10 (23.3)	9 (20.9)	
Genetic	23 (59.0)	13 (33.3)	3 (7.7)	
Severity of neuromuscular disease^Ɨ^				0.007*
Low	21 (72.4)	6 (20.7)	2 (6.9)	
Moderate	22 (61.1)	8 (22.2)	6 (16.7)	
High	3 (18.8)	9 (56.2)	4 (25.0)	
Mobility				0.015*
Walk	40 (70.2)	12 (21.1)	5 (8.8)	
Walk with help	5 (41.7)	4 (33.3)	3 (25.0)	
Wheelchair	3 (25.0)	6 (50.0)	3 (25.0)	
Gender				0.031*
Men	14 (41.2)	14 (41.2)	6 (17.6)	
Women	34 (69.4)	9 (18.4)	6 (12.2)	
Age > 65 yrs old	9 (47.4)	9 (47.4)	1 (5.3)	0.070
Respiratory failure	12 (42.9)	10 (35.7)	6 (21.4)	0.079
Heart failure	8 (50.0)	6 (37.5)	2 (12.5)	0.617
Obesity	2 (25.0)	4 (50.0)	2 (25.0)	0.097
Hypertension	1 (9.1)	7 (63.6)	3 (27.3)	0.001*
Diabetes	1 (10.0)	4 (40.0)	5 (50.0)	0.001*
Corticosteroids^‡,^^§^	6 (46.2)	4 (30.8)	3 (23.1)	0.670
Immunosuppressants other than corticosteroids^‡,^^§^	8 (50.0)	4 (25.0)	4 (25.0)	0.764

*n* number of cases, *No. (%)* number of cases (% of cases)

^Ɨ^According to the physician

^‡^Prior to COVID-19

^§^Statistical analysis have been performed only on populations with acquired diseases

*p < 0.05 was statistically significant

Univariate analyses showed that the severity of NMD (p = 0.007), high arterial blood pressure (p = 0.001), diabetes (p = 0.001), degree of mobility (p = 0.015) and gender (p = 0.031) were significantly associated with the degree of severity of COVID-19 for NMD patients (Table 4).

These variables, along with obesity (p = 0.097), age > 65 years (p = 0.070) and type of neuromuscular disease (p = 0.095) were included in a multinomial logistic regression (except for respiratory failure due to small numbers, which were insufficient to enable the model to converge to a final solution). Compared to the reference category “low COVID-19 severity”, multinomial logistic regression showed that patients with severe NMD (OR = 10.840; 95% CI 1.888–62.255, p = 0.008) and patients with hypertension (OR = 15.511; 95% CI 1.526–157.619, p = 0.020) had a higher risk of developing moderate COVID-19 severity. In addition, patients with severe NMD (OR = 11.154; 95% CI 1.042–119.379, p = 0.046) and patients with diabetes (OR = 14.993; 95% CI 1.292–174.014, p = 0.030) had a higher risk of developing high COVID-19 severity.

Concerning medication, statistical analysis of the population suffering from acquired and non-hereditary forms of NMD showed that corticosteroids and other immunosuppressants were not significantly associated with the degree of COVID-19 severity (p = 0.670 and p = 0.764 respectively).

## Discussion

This multicenter study showed that 84 patients with NMD followed by the French expert centers network contracted COVID-19 during the period from March 25, 2020 to May 11, 2020. Our cohort included 34 patients with myasthenia gravis, 23 with neuropathy and 27 with myopathy. COVID-19 caused an aggravation of their NMD in 26 (31%) patients and death in 9 (11%) patients. COVID-19 had no effect on 48 (58%) of them. Our study showed that the impact of COVID-19 on NMD did not depend on the category (neuropathy, myopathy and myasthenia gravis), acquired/hereditary nature of NMD or severity of NMD. Diabetic patients with NMD were at a greater risk of dying from COVID-19. In this cohort, COVID-19 severity was considered to be low in 48 (58%) NMD patients. Risk factors associated with the degree of COVID-19 severity were the severity of NMD, hypertension and diabetes. In our cohort, corticosteroids and other immunosuppressants were not found to be factors associated with the degree of COVID-19 severity.

The state of health (cardio-respiratory failure, immunosuppressive medication, and other comorbidities such as diabetes or hypertension) of patients suffering from NMD has caused increased fear of COVID-19 having a devastating impact on this population. Although significant vigilance should be maintained, this fear was somewhat alleviated when we compared the data from this study with impact of COVID-19 on the general population. Indeed, on May 11, 2020, in the context of the onset of a health crisis at a time when there were few PCR tests, France reported 178 719 positive cases including 26 646 deaths, 19 572 people hospitalized in COVID-19 units and 2 712 in ICU [8]. The prevalence of COVID-19 in the population of NMD patients was therefore slightly lower than that of the French general population (0.0017 for NMD patients versus 0.0026 for the general population). The death rate in the NMD patient population was also lower than that of the French general population (0.11 vs. 0.14, respectively). However, the rate of hospitalization in a medical unit and in ICU was higher in the NMD patient population than in the French general population (medical unit hospitalization: 0.27 for NMD patients, 0.14 for the general population; intensive care: 0.14 for NMD patients, 0.019 for the general population). Interestingly, other epidemiological studies have been performed on French cohorts of patients with multiple sclerosis (MS) and patients with rheumatic and inflammatory diseases (iRMD) [9, 10]. In these diseases, the death rate was lower than in our study (3.5% for MS and 8% for iRMD respectively, vs 11% in our cohort), similarly to the hospitalization rate (21% for MS and 36% for iRMD respectively, vs. 42% in our cohort, medical unit and ICU). Other large epidemiological studies will be required to confirm these results.

Previous studies have reported that obesity, hypertension, diabetes, gender, immunosuppressive drugs/corticosteroids and age could be risk factors playing an important role in the evolution of COVID-19 in the general population [4, 5, 11]. As expected, we found that the risk factors for the general population were identified as risk factors for the impact or degree of severity of COVID-19 in NMD patients. Interestingly, hypertension, gender and diabetes have also been recognized as common risk factors associated with COVID-19 severity in MS or iRMD cohorts [9, 10].

However, in our cohort of acquired NMD patients, therapy with corticosteroids or other immunosuppressants did not appear to be risk factors for severe forms of COVID-19. In accordance with this, a study performed on 15 myasthenia gravis patients infected with COVID-19 showed that immunosuppressive therapies did not seem to cause any additional unfavorable outcome [12]. Likewise, data from myasthenic patients in our cohort showed that corticosteroids or other immunosuppressants used in the management of myasthenia gravis were not a risk factor for poorer outcomes [13]. However, studies performed on a cohort of 694 iRMD patients showed that long-term corticosteroid intake increased the risk of developing severe forms of COVID-19 [10]. The outcome of medication on COVID-19 severity could depend on doses, overlap between medications and/or timing between medication and infection. For these reasons, results on corticosteroids/other immunosuppressants should be interpreted with caution. Indeed, in our study, the sample size of NMD patients with COVID-19 receiving corticosteroids/other immunosuppressants was small and cases were heterogeneous, concerning severity of the disease, type of pathology, type of drug administered and doses. Currently, management of immunosuppressive/immune-modulator medication in this health crisis remains a challenge.

Similarly to scientific literature [14], our study showed that few patients with NMD contracted COVID-19 in France during this period. All of these results could be explained by patients paying particular attention to self-isolation and hygiene measures, working from home more often and actions carried out by neuromuscular patient organizations and Filnemus, whose ambition during this public health crisis was to fight this virus and to protect patients with neuromuscular diseases. Nonetheless, one of major deleterious impacts of COVID-19 is that the health crisis has had huge consequences on the management of NMD patients [14–16]. Several patients have foregone or did not have access to medical care and have developed more stiffness, more pain or have reduced their walking distance.

In the context of an emergency health crisis, this dynamic observatory of the number and state of health of patients has enabled Filnemus to directly monitor the evolution of this virus in patients with NMDs. Knowing the evolution and the impact of this virus on NMD patients enabled Filnemus to draw up a document intended to help resuscitators in their decision-making, in order to provide equal opportunities for the management of patients with NMD in intensive care. The data collected in this survey is essential to understand the impact of COVID-19 on this population but remains insufficient. Although our method of data collection was effective it was not exhaustive. One of the limitations of our study is the lack of confirmation of COVID-19 for several patients in our cohort due to a lack of testing at that time. Indeed, many cases with symptoms suggestive of COVID-19 could not be confirmed by PCR test or Thoracic CT-scan and asymptomatic cases were not detected. Although the clinical diagnosis allowing patients to be included in the cohort is based on very specific criteria published in major scientific journals and which are highly suggestive of COVID-19, additional studies will help deepen knowledge on this subject and clarify the impact of this virus on various NMDs.

The aim of this study was to evaluate the consequences of COVID-19 on NMD patients in the short term. Based on our results we cannot exclude that the effects of COVID-19 on these patients could appear in the longer term. It would be interesting to investigate this as a second step.

To conclude, this French multicenter study shows that few patients with NMDs presented symptoms of and/or tested positive for COVID-19 during this first lockdown period. COVID-19 in patients with NMD did not systematically lead to NMD aggravation and severe forms of COVID-19. Diabetes is a risk factor for unfavorable outcome for NMD − COVID-19 + patients. The severity of COVID-19 for NMD patients depended on the degree of NMD severity, diabetes and hypertension. Finally, in this cohort, treatment with corticosteroids or other immunosuppressants was not associated with higher COVID-19 severity. As a whole, these results should help adapt the management of neuromuscular patients during the COVID-19 pandemic and also the prioritization and strategy of vaccination especially for the most severely ill patients.

## Methods

### Data collection

Data was collected in real time by physicians from 71 expert centers belonging to Filnemus. Expert centers and patient organizations made calls to 18 000 NMD patients to ensure their good health. Patients presenting COVID-19 clinical symptoms were encouraged to contact their physician. The survey was performed during the first French lockdown period that lasted from March 25, 2020 to May 11, 2020. This anonymous study was carried out in accordance with good clinical practice, General Data Protection Regulations (GDPR) and the local ethics committee. Patients included in this study were informed by their physician who signed informed consent notes of non-objection. All the patients in this study had NMD and were strongly suspected to be infected by SARS-CoV2. COVID-19 was confirmed either by PCR, or suspected by thoracic computed tomography (thoracic CT-Scan) [17] or symptomatology including several associated signs suggesting COVID-19: cough, fever, hyposmia, skin manifestations, myalgia, headache, diarrhea, breathlessness, chest tightness and anosmia. At this time in the pandemic PCR testing was not performed systematically. Patients with clinical signs highly suggestive of COVID-19 but without positive PCR or thoracic CT-Scan results were considered to be probable cases.

### Variables studied

Data collected prior to COVID-19 were: age, gender, diagnosis, acquired/hereditary form of disease, degree of mobility (walking without help, walking with help or in a wheelchair), comorbidities, presence of respiratory (yes or no) or cardiac (yes or no) failure, degree of severity of the NMD according to the physician (low, moderate or severe) and medication. Categorization of the severity of patients' neuromuscular disease was discussed during the Filnemus COVID-19 meetings that have been held weekly since the beginning of the pandemic and a consensus was reached.

Data related to COVID-19 were: diagnosis of COVID-19 (PCR/thoracic CT-Scan or clinical diagnostic criteria), impact of COVID-19 on the disease (no effect, aggravating factor, or death), and categories of medical care (home care, medical unit, ICU). The severity of COVID-19 was considered low for patients with home care, moderate for patients needing hospitalization in a medical unit and high for patients hospitalized in ICU.

The hospitalization rate was the ratio of NMD patients hospitalized in a medical unit over the total number of patients infected by SARS-CoV2 virus. The ICU rate was determined by the number of NMD patients hospitalized in ICU divided by the total number of NMD patients infected by SARS-CoV2 virus. The death rate was calculated as the number of NMD patients dying after SARS-CoV2 infection divided by the total number of NMD patients infected by SARS-CoV2. The prevalence of COVID-19 in the NMD population was defined as the number of NMD patients infected by SARS-CoV2 divided by the total number of NMD patients in France.

### Outcomes

The primary endpoint was the impact of COVID-19 on NMD. The effect of COVID-19 was classified into three levels according to the physician’s evaluation: no effect, aggravating factor, death. The secondary endpoint was the impact of NMD on COVID-19 severity. COVID-19 severity was assessed and classified according to the medical care received by each patient: low COVID-19 severity = home care, moderate COVID-19 severity = hospitalization in a medical unit, high COVID-19 severity = hospitalization in ICU.

### Statistical analysis

The basic characteristics of the cohort were summarized as frequencies and percentages for categorical variables and were expressed as mean ± standard deviation for normally distributed continuous variables.

Univariate and multivariate analyses were performed on the cohort. First, in the univariate analysis, risk factors were assessed using analysis of variance (ANOVA) to compare continuous variables across groups, and Chi-square test or Fisher-Freeman-Halton’s exact test were used for categorical variables, as appropriate. Second, two stepwise multinomial logistic regressions were used for the multivariate analysis by selecting all variables with a p-value not exceeding 0.15 in the univariate analysis to determine the independent risk factors for the effect of COVID-19 on NMD (Model 1, "no effect" reference category) and for the effect of NMD on the severity of COVID-19 (Model 2, “low COVID-19 severity” reference category). Odds ratios (OR) with their associated 95% confidence intervals (CI) were reported. A p-value < 0.05 was considered statistically significant. All statistical analyses were performed using SPSS software, version 20.0 (SPSS, Inc., Chicago, Illinois).

## Data Availability

The datasets used and/or analyzed during the current study are available from the corresponding author on reasonable request.

## Abbreviations

BNDMR: National Data Bank of Rare Diseases
CI: Confidence interval
COVID-19: Coronavirus disease 2019
FILNEMUS: French Health Care Network for Rare Neuromuscular Diseases
GDPR: General Data Protection Regulations
ICU: Intensive care unit
iRMD: Rheumatic and inflammatory diseases
MS: Multiple sclerosis
NMD: Neuromuscular diseases
OR: Odds ratio
Thoracic CT-Scan: Thoracic computed tomography
